# Correction: A Comparison of Peak Callers Used for DNase-Seq Data

**DOI:** 10.1371/journal.pone.0105136

**Published:** 2014-08-05

**Authors:** The PLOS ONE Staff

There are errors in the author affiliations. The affiliations should appear as shown here:

Hashem Koohy 1,2, Thomas A. Down 1, Mikhail Spivakov 1, Tim Hubbard 2

1 The Babraham Institute, Babraham Research Campus, Cambridge United Kingdom

2 Wellcome Trust Sanger Institute, Wellcome Trust Research Campus, Cambridge, United Kingdom.
