# Supplementary figures and images for: A Comparison of Peak Callers Used for DNase-Seq Data
Source: PLoS One. 2014 May 8;9(5):e96303. doi: 10.1371/journal.pone.0096303 (PMC4014496; doi:10.1371/journal.pone.0096303)

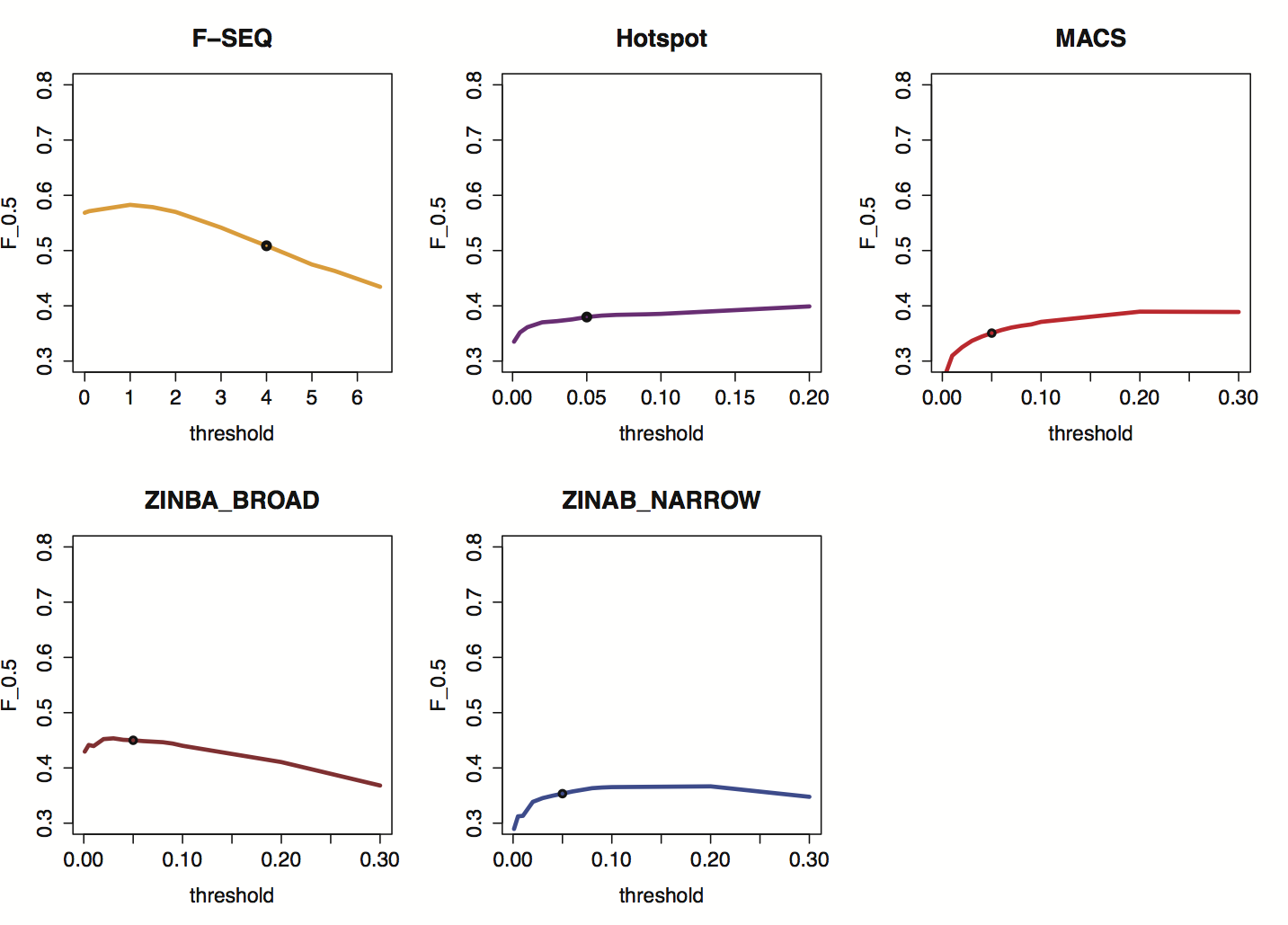

Supplement: Figure S1 — Performance of Algorithms Over One Cell Type From the “End Capture” Protocol. Similar to Figure 6, the performance of each algorithm was evaluated using GM12878 cell type obtained from Duke University “end capture” protocol [26]. (TIFF) [file pone.0096303.s001.tif]

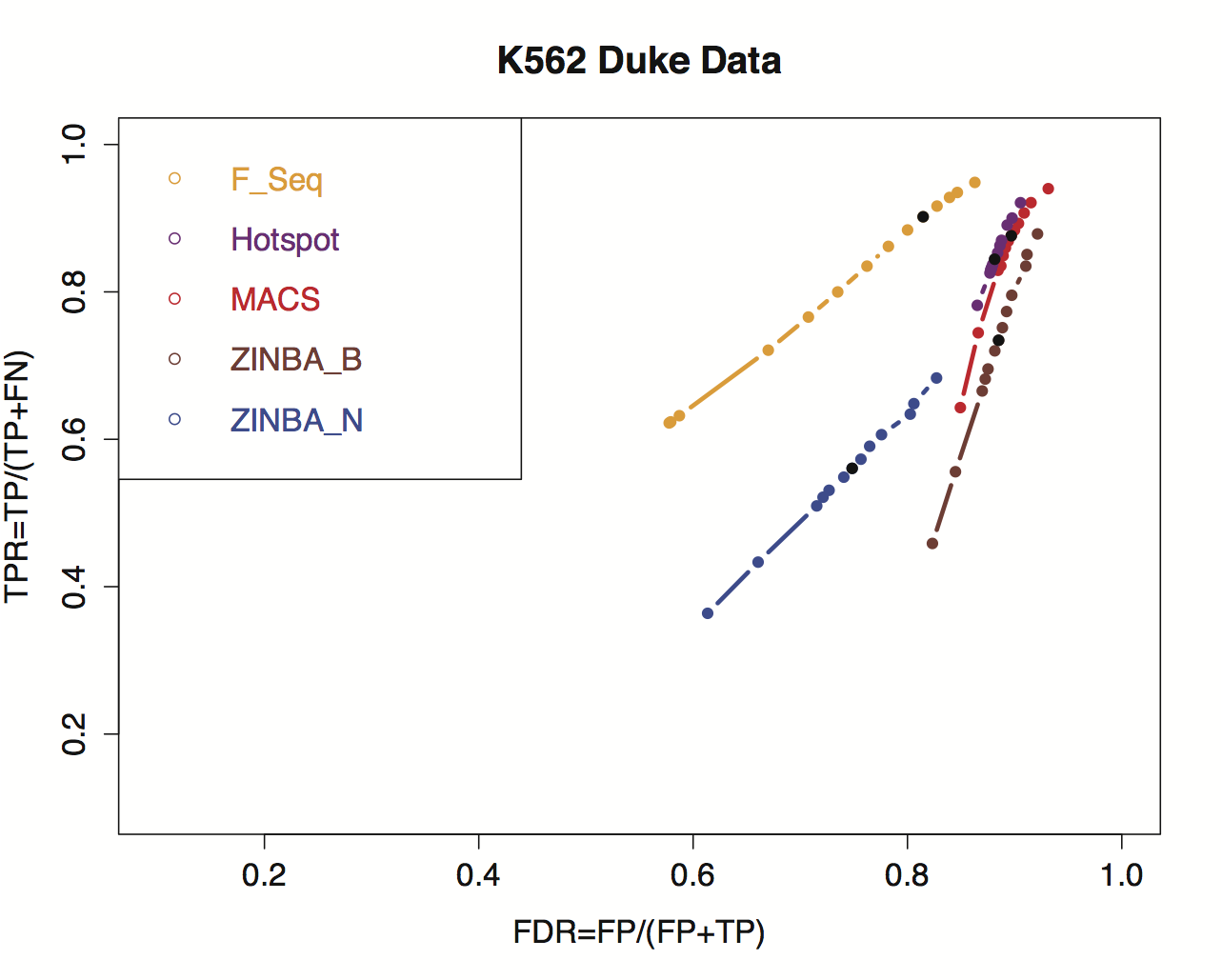

Supplement: Figure S2 — Comparison of TPR and FDR of Peak Callers with “End Capture” Data. Depicted here is the result of our comparison of four algorithms over data obtained from Duke University end capture protocol. (TIFF) [file pone.0096303.s002.tif]

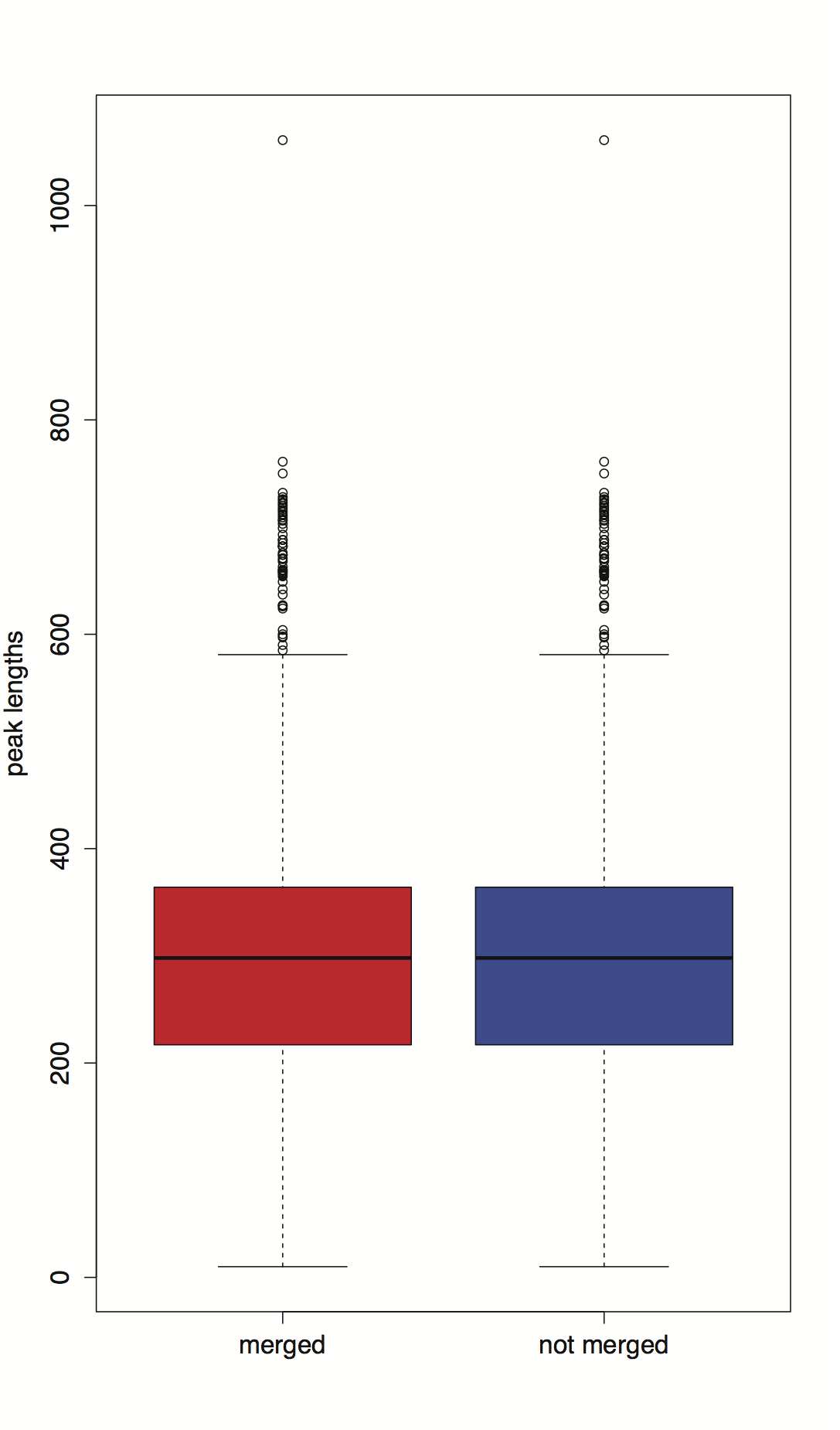

Supplement: Figure S3 — Effect of Hotspot “merge” Parameter on the Distribution of Peak Lengths. Distribution of Hotspot peak length merged (default: peaks closer than 150 bp are merged) versus not merged in UW K562 cells. (TIFF) [file pone.0096303.s003.tif]

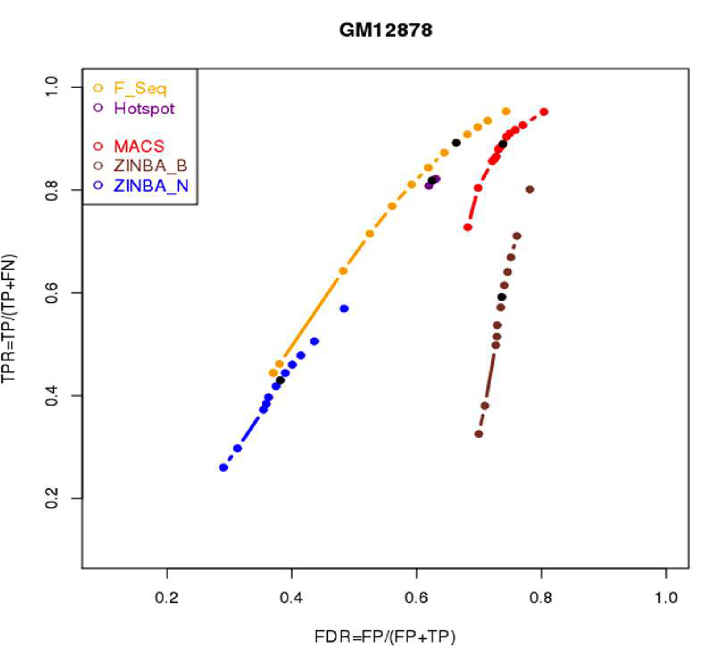

Supplement: Figure S4 — Effect of Hotspot “zscore” Parameter on its Performance. Hotspot was run at a range of z-score threshold ranging from to and all other parameters were kept as default. The other three algorithms were also run at a range of signal threshold (as described in main text). (TIFF) [file pone.0096303.s004.tif]

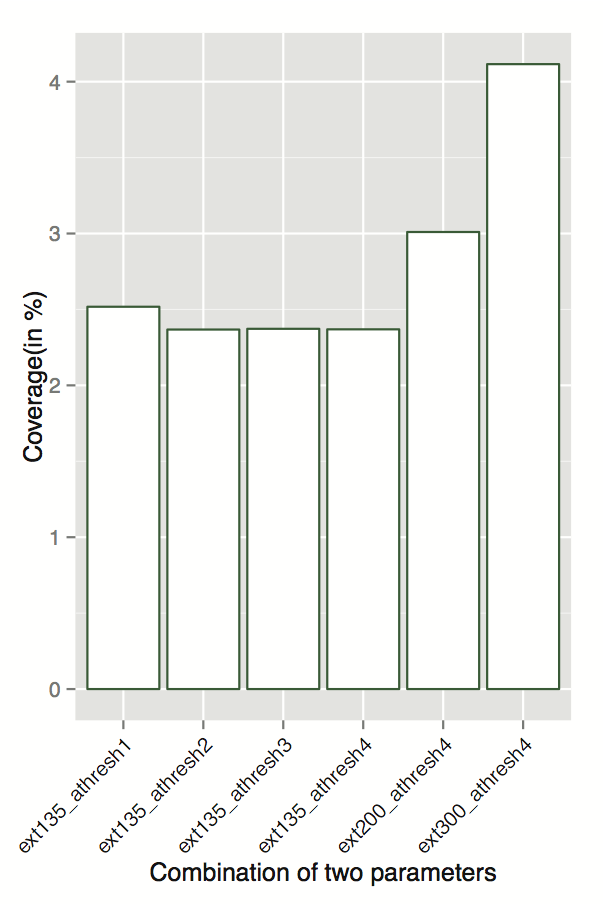

Supplement: Figure S5 — Effect of the Number of Hits and Extension on ZINBA Coverage. Depicted here is the coverage (as defined in main text) of ZINBA when run at various combinations of number of hits per read known as “athreshold”(run at values equal to 1, 2, 3, 4) and the average of fragment lengths known as “extension”(run at values equal to 135, 200 and 300 bp). (TIFF) [file pone.0096303.s005.tif]

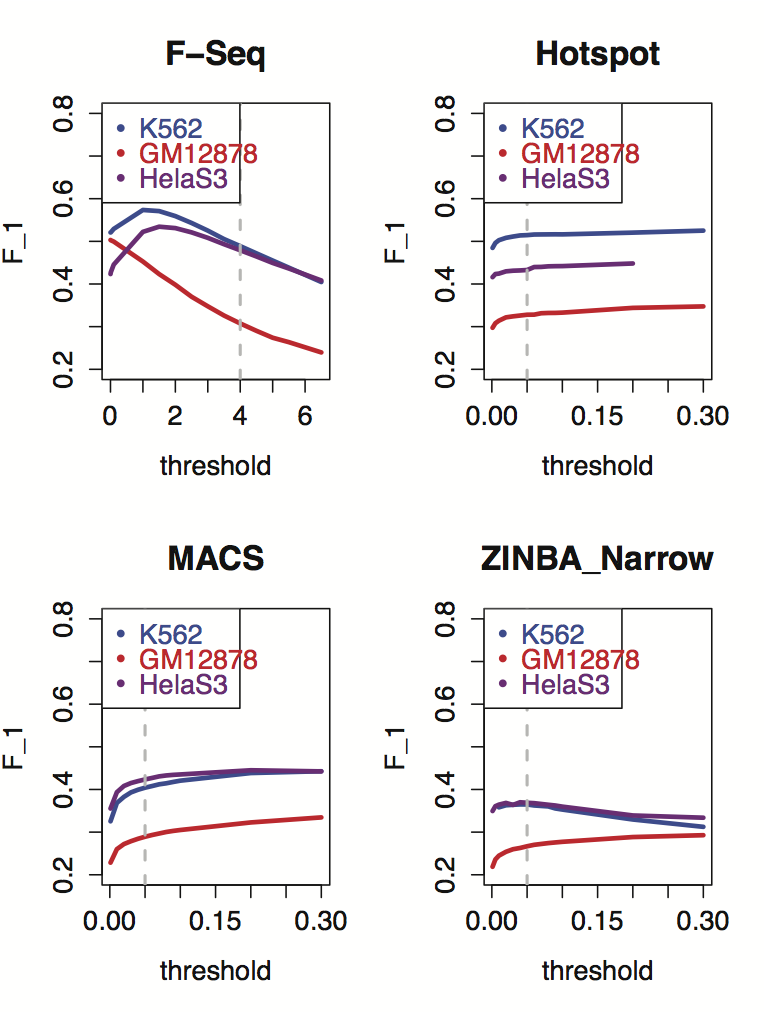

Supplement: Figure S6 — The F-scores of the Algorithms Across the Three Cell Types Assuming . Illustrated here is the data shown in Figure 6, but computed assuming the parameter equal to 1, which corresponds to same weight associated with both sensitivity and specificity. The vertical dash lines show the default threshold values in each algorithm. (TIFF) [file pone.0096303.s006.tif]
